# High resolution 3-Dimensional imaging of the human cardiac conduction system from microanatomy to mathematical modeling

**DOI:** 10.1038/s41598-017-07694-8

**Published:** 2017-08-03

**Authors:** Robert S. Stephenson, Andrew Atkinson, Petros Kottas, Filip Perde, Fatemeh Jafarzadeh, Mike Bateman, Paul A. Iaizzo, Jichao Zhao, Henggui Zhang, Robert H. Anderson, Jonathan C. Jarvis, Halina Dobrzynski

**Affiliations:** 1 Comparative Medicine Lab, Department of Clinical Medicine, Aarhus University, Aarhus, Denmark; 2 School of Sport and Exercise Sciences, Liverpool John Moores University, Liverpool, UK; 3 Faculty of Biology, Medicine and Health, University of Manchester, Manchester, UK; 4 School of Physics and Astronomy, University of Manchester, Manchester, UK; 5 National Institute of Legal Medicine, Bucharest, Romania; 6 The Visible Heart Laboratory, University of Minnesota, Minneapolis, USA; 7 Auckland Bioengineering Institute, University of Auckland, Auckland, New Zealand; 8 Institute of Genetic Medicine, University of Newcastle, Newcastle, UK

## Abstract

Cardiac arrhythmias and conduction disturbances are accompanied by structural remodelling of the specialised cardiomyocytes known collectively as the cardiac conduction system. Here, using contrast enhanced micro-computed tomography, we present, in attitudinally appropriate fashion, the first 3-dimensional representations of the cardiac conduction system within the intact human heart. We show that cardiomyocyte orientation can be extracted from these datasets at spatial resolutions approaching the single cell. These data show that commonly accepted anatomical representations are oversimplified. We have incorporated the high-resolution anatomical data into mathematical simulations of cardiac electrical depolarisation. The data presented should have multidisciplinary impact. Since the rate of depolarisation is dictated by cardiac microstructure, and the precise orientation of the cardiomyocytes, our data should improve the fidelity of mathematical models. By showing the precise 3-dimensional relationships between the cardiac conduction system and surrounding structures, we provide new insights relevant to valvar replacement surgery and ablation therapies. We also offer a practical method for investigation of remodelling in disease, and thus, virtual pathology and archiving. Such data presented as 3D images or 3D printed models, will inform discussions between medical teams and their patients, and aid the education of medical and surgical trainees.

## Introduction

The specialised cardiomyocytes, known as the cardiac conduction system, are anatomically discrete from the working myocardium, and cannot be visualised using traditional non-invasive techniques. They have the ability spontaneously to generate electrical impulses and to function as pacemakers^1, 2^. They act also to conduct impulses throughout the heart, in a rapid and coordinated fashion^1^. The main components of the cardiac conduction system are the sinus node of the right atrium, which is the primary pacemaker; the atrioventricular conduction axis, which normally forms the only pathway allowing excitation to pass between the atria and ventricles; and the Purkinje network, which allows fast and coordinated conduction within the ventricles^3, 4^.

Cardiac arrhythmias and conduction disturbances affect 0.1% of adults younger than 55 years old, increasing to 9% in those older than 80 years^5–8^. They also occur in almost 40% of patients attending cardiology outpatient clinics^7^. The onset of such conditions may be attributed to age, genetic predisposition, lifestyle, myocardial remodelling, fibrosis of tissues, and history of surgical procedures. We have shown previously the 3D distribution of the cardiac conduction system in animal hearts^9–12^, along with the link between its molecular and micro-anatomical remodelling in disease^13^. Knowledge of the 3D micro-anatomy of the human cardiac conduction system, therefore, is crucial. Up to now, this information has been absent from the literature.

Biophysically-detailed mathematical models are powerful tools with which to investigate normal and pathological cardiac conduction. Efforts have been made to produce detailed electrophysiological data to inform such models^14–17^. The fidelity of the current models, however, is compromised by the use of simplified geometric data sets from disparate species^16, 18–20^. In order to produce simulations with the highest possible structural fidelity, models must include; (1) accurate morphological representations of the human cardiac conduction system and its interface with the working myocardium, and (2) the 3D orientation of the cardiomyocytes within these tissues. Individual cardiomyocytes are of the order of a few tens of microns in dimension, and the path and velocity of conduction is known to be dictated by their orientation^21^. Depiction of 3D cellular alignment, therefore, should ideally be generated from image data as close as possible to this order of spatial resolution.

Our present understanding of sinus node and atrioventricular node morphology owes much to painstaking reconstruction of 2D serial histology^22–27^. Any such technique is limited in 3D resolution by the distance between successive sections, typically 60–340 µm^23, 25^. Such destructive analysis is further limited by the requirement to use preparations of an isolated part of a given heart, small enough for routine fixation and sectioning. This makes the placement of the analysed volumes back into the context of the whole heart difficult, and prone to error. As a result, the conduction system is rarely presented in the attitudinally correct position^28^. Despite these caveats, we have shown previously that histology and immunohistochemistry remain crucial techniques to validate 3D imaging^2, 12, 13^.

Current histological depictions of the orientation of cardiomyocytes commonly suffer from poor z-plane resolution and inaccurate registrations. Diffusion tensor magnetic resonance imaging has become the gold standard for investigation of the relative orientation of cardiomyocytes^29–32^. However, because of its somewhat gross spatial resolution, namely ~600–3,000 um^3^, it produces a representation of mean cellular orientation. Such a technique cannot, therefore, show the complex 3D arrangement of the individual chains of cardiomyocytes.

The scientific literature and medical textbooks provide, therefore, mixed, and often inaccurate, descriptions of both the cardiac conduction system and the arrangement of cardiomyocytes. For instance, the structure and function of the paranodal region is still contested^14, 22, 33–35^, there is a common misconception that the Purkinje network runs exclusively subendocardially, and many believe the chains of cardiomyocytes can be unravelled as a continuous and ordered helical myocardial band^2, 36^. In order to provide definitive answers to these existing controversies, there is the need to present the micro-structure of the whole heart in 3-dimensions at cellular resolution. Micro-computed tomography (micro-CT) can now provide such a dataset from an intact heart using scan times below 1 hour^12, 37^.

Here, we present, to the best of our knowledge, the first 3D representation of the cardiac conduction system within an *ex-vivo* intact human heart, and place them in the attitudinally correct position. Data was obtained by means of the non-invasive, non-destructive imaging technique known as contrast enhanced micro-CT. We have resolved the relative extent of the sinus node, its projections, and the associated paranodal area. We have shown that cellular orientation can be extracted from a high-resolution whole heart data set, demonstrating the myocardium to be a complex mesh-work of cardiomyocyte chains. This technique will facilitate an improved understanding of the altered morphology of the cardiac conduction system in congenital malformations, heart disease, and ageing. This new level of geometric detail will also improve the fidelity of mathematical models, providing new insights into the links between morphological remodelling and the manifestation of arrhythmias and conduction disturbances in humans.

## Results

We present accurate 3D representations of the location of the cardiac conduction system in the intact human heart using the non-destructive imaging technique of contrast enhanced micro-CT. Contrast enhancement permits us to distinguish the relevant tissue types based on their differential attenuation of the x-ray beam. As a result of iodine infusion; fat, working myocardium, specialised nodal tissue, paranodal tissue, and connective tissue exhibit decreasing levels of attenuation respectively, and thus decreasing voxel values in the tomographic data. We have discussed previously the possible mechanism behind the observed differential uptake of iodine by biological tissues^12, 38^. The spatial resolution obtained was also sufficient to demonstrate cardiomyocyte orientation, thus permitting us to produce an anatomically and biophysically detailed mathematical model of cardiac electrical activation.

### The sinus node and its paranodal area

**Figure 1:**
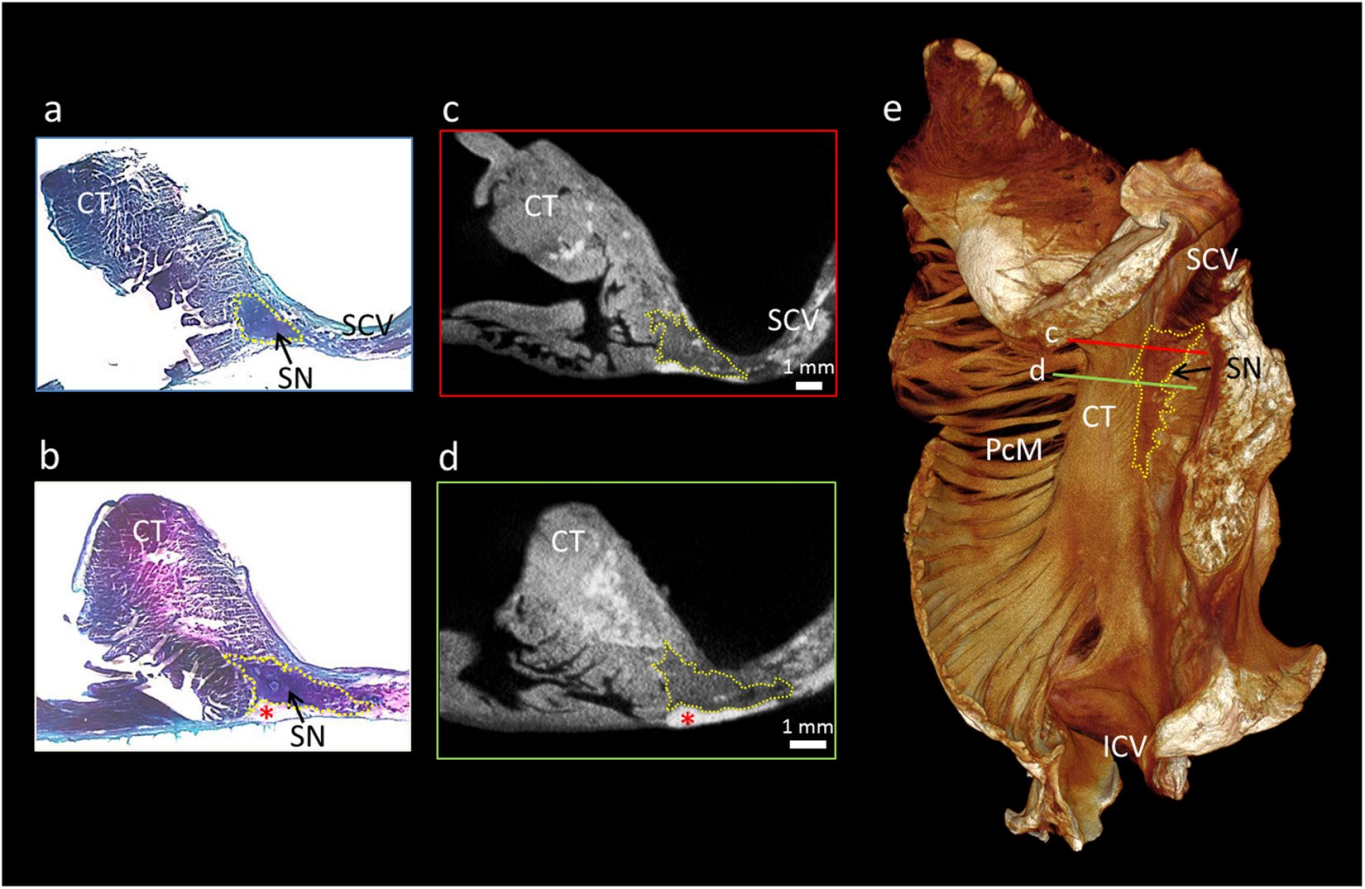
Micro-CT allows objective discrimination of the human sinus node. This figure demonstrates high resolution (28 × 28 × 28 µm^3^) micro-CT data from part of the right atrium containing the sinus node. For this figure, the sinus node is outlined in yellow in short-axis micro-CT sections (**c**,**d**) and in matching histological sections taken from the same sample (**a**,**b**). The plane of section in c and d is shown on the 3D volume rendering (endocardial view) (**e**). The sections are available to view without the outlines in the supplementary Fig. S2. CT- terminal crest, ICV- inferior caval vein, PcM- pectinate muscles, SCV- superior caval vein, SN- sinus node, *- indicates epicardial fat.

**Figure 2:**
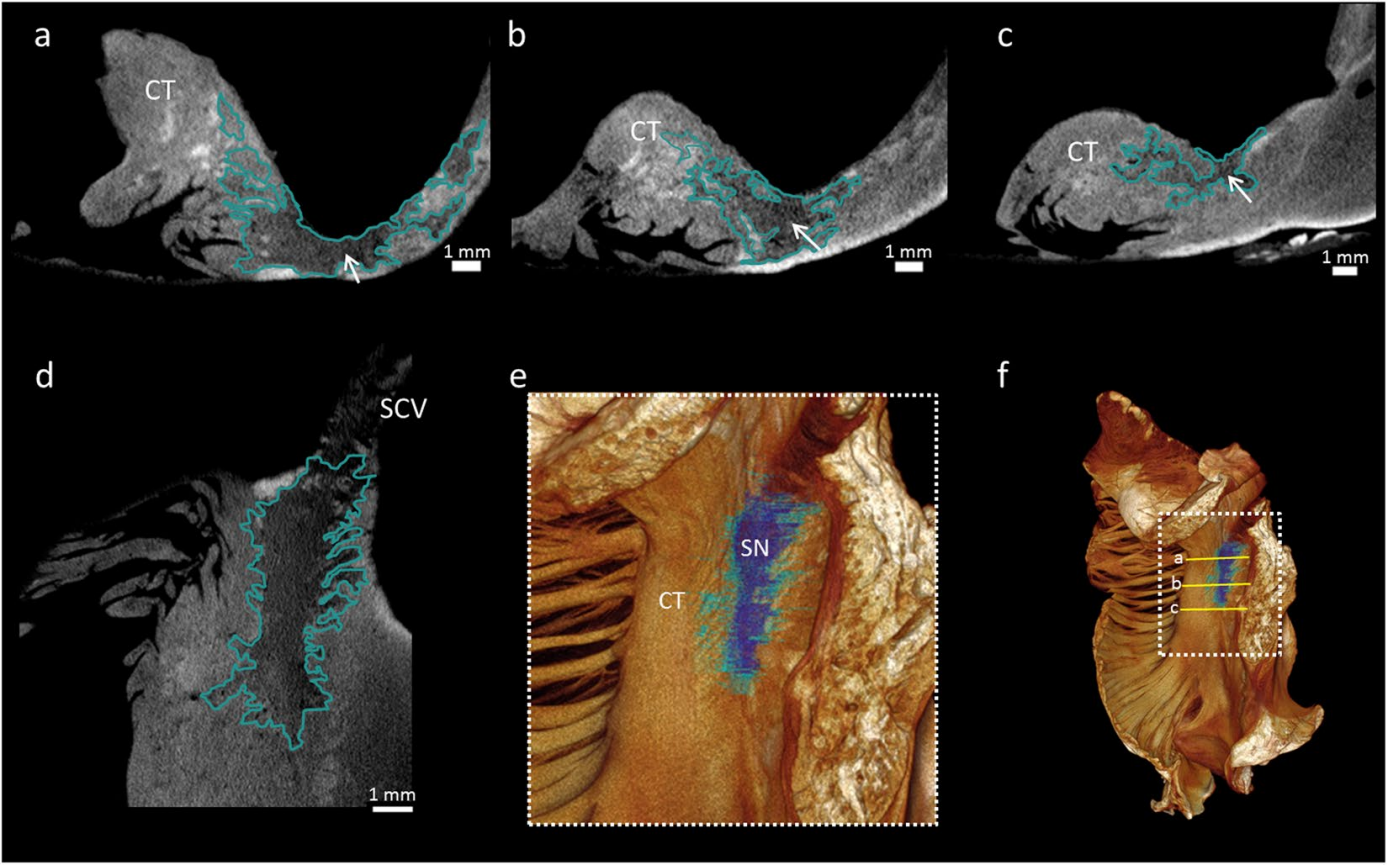
Objective segmentation of the human sinus node and its paranodal area. This figure demonstrates high resolution (28 × 28 × 28 µm^3^) micro-CT data from part of the right atrium containing the sinus node. The extent of the sinus node and its paranodal area is outlined in short-axis (**a**–**c**) and long-axis (**d**) micro-CT images. (**e**) Volume rendering (endocardial view) showing objective segmentation of the low pixel values corresponding to the body of the sinus node in dark blue; pixel values corresponding to the paranodal area are shown in turquoise. The plane of section in a-c is shown on the 3D volume rendering (**f**). Arrows indicate the approximate seed point for the objective segmentation of the sinus node. Sinus node body length = 14.8mm, width = 4.3mm. The sections are available to view without the outlines in the supplementary Fig. S2. CT- terminal crest, SN- sinus node.

The serial short-axis 2D tomograms, along with their 3D volume renderings, show the primary pacemaker of the heart, the sinus node, to have a complex 3D shape with many radiating projections (Figs 1 and 2, Supplementary Figs S1 and S2). The node narrows at the superior cavoatrial junction, and tapers caudally as it extends towards the inferior caval vein (Fig. 1 and Supplementary Fig. S1). In some places the node is enclosed by the surrounding myocardium and epicardial fat. In other regions it extends close to the epicardial and endocardial surfaces (Figs 1 and 2, Supplementary Figs S1 and S2). Using automatic segmentation (see data supplement), we were able to reconstruct the node in fine detail (Fig. 2). The main body of the node (Fig. 2 length = 14.8mm, width = 4.3mm) was housed within the intercaval region, but islands and projections of nodal tissue were also observed within the terminal crest (also referred to as the crista terminalis), extending towards the epicardial terminal groove, the pectinate muscles, and towards the interatrial septum (Fig. 2 and Supplementary Figs S1 and S2). These regions, previously described as paranodal, could also be differentiated from the working myocardium in the micro-CT data, appearing as lower attenuating regions. Their voxel values, however, were higher than those found in the main body of the sinus node. In Fig. 2e and f we show a 3D segmentation of the sinus node produced by automatic segmentation. The blue colour indicates voxels selected using automatic segmentation, with the sinus node body as the seed point. Turquoise indicates automatic segmentation of adjacent projections and islands, which have pixel values higher than those of the sinus node, but lower than the working myocardium. Serial tomograms representing the data shown in Fig. 2 are presented in supplementary Fig. S2, along with an epicardial volume rendering showing the interaction of these projections with the terminal crest and adjoining pectinate muscles (Supplementary Fig. S1).

### The atrioventricular conduction axis

**Figure 3:**
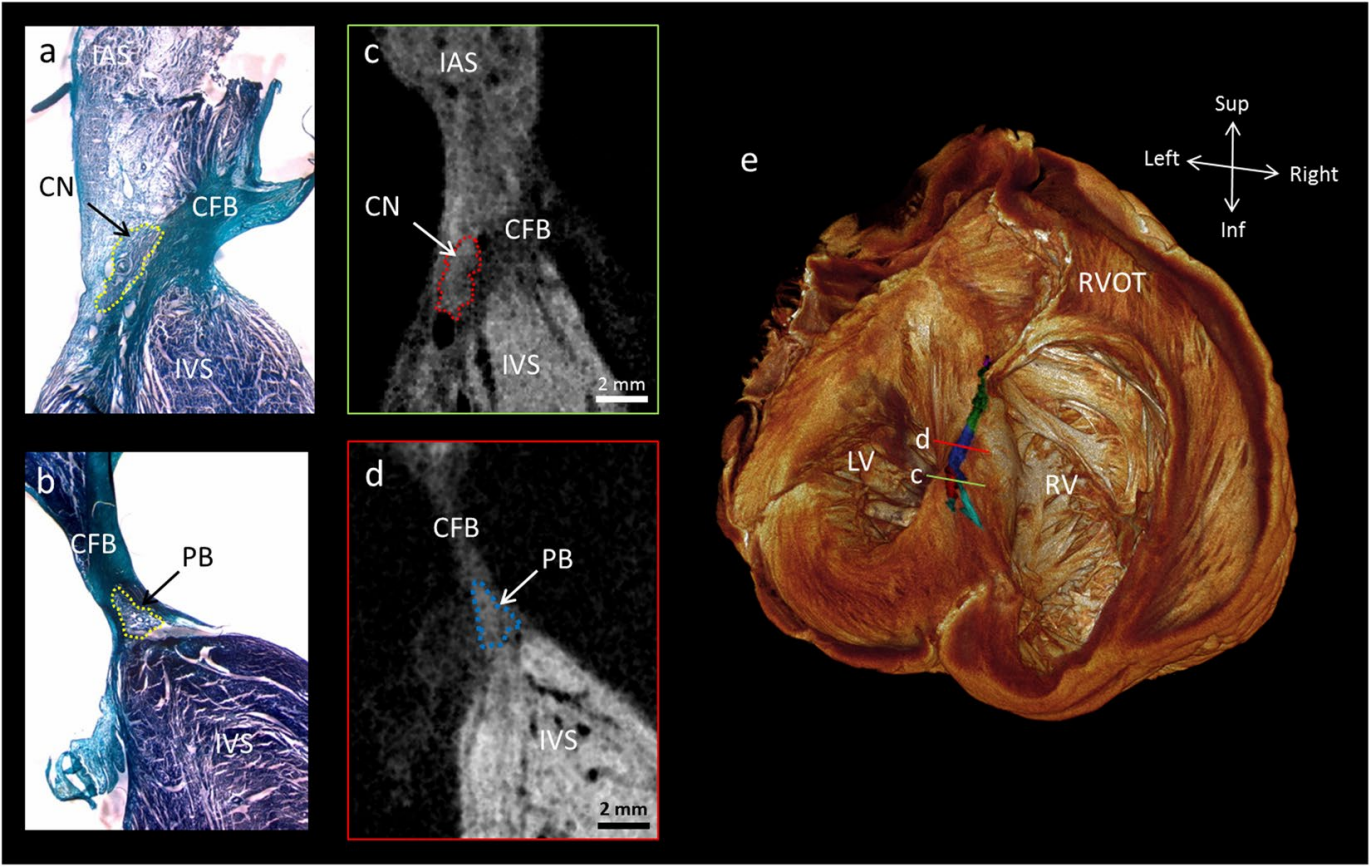
The human atrioventricular conduction axis resolved by micro-CT. This figure demonstrates high resolution (73 × 73 × 73 µm^3^) micro-CT data from a whole human heart. It shows the 3D extent and location of the segmented atrioventricular conduction axis across the upper surface of the interventricular septum (**e**). The viewpoint in e is from the atria, looking down into the ventricular chambers. Illustrative long-axis sections from the micro-CT dataset and from corresponding histological sections show the compact node in the right atrium proximally and inferiorly (**a**,**c**), becoming the penetrating bundle (**b**,**d**), and extending anteriorly and slightly rostrally to become the branching bundle (**e**). The plane of section in c and d is shown on the 3D volume rendering (**e**). The sections are available to view without the outlines in the supplementary Fig. S3. Atrioventricular conduction axis colour coding in panel e; turquoise- inferior nodal extension, red- compact node, blue- penetrating bundle, green- branching bundle, purple- dead-end tract. CFB- central fibrous body, CN- compact node, IAS- interatrial septum, IVS- interventricular septum, LV- left ventricle, PB- penetrating bundle, RV- right ventricle, RVOT- right ventricular outflow tract.

**Figure 4:**
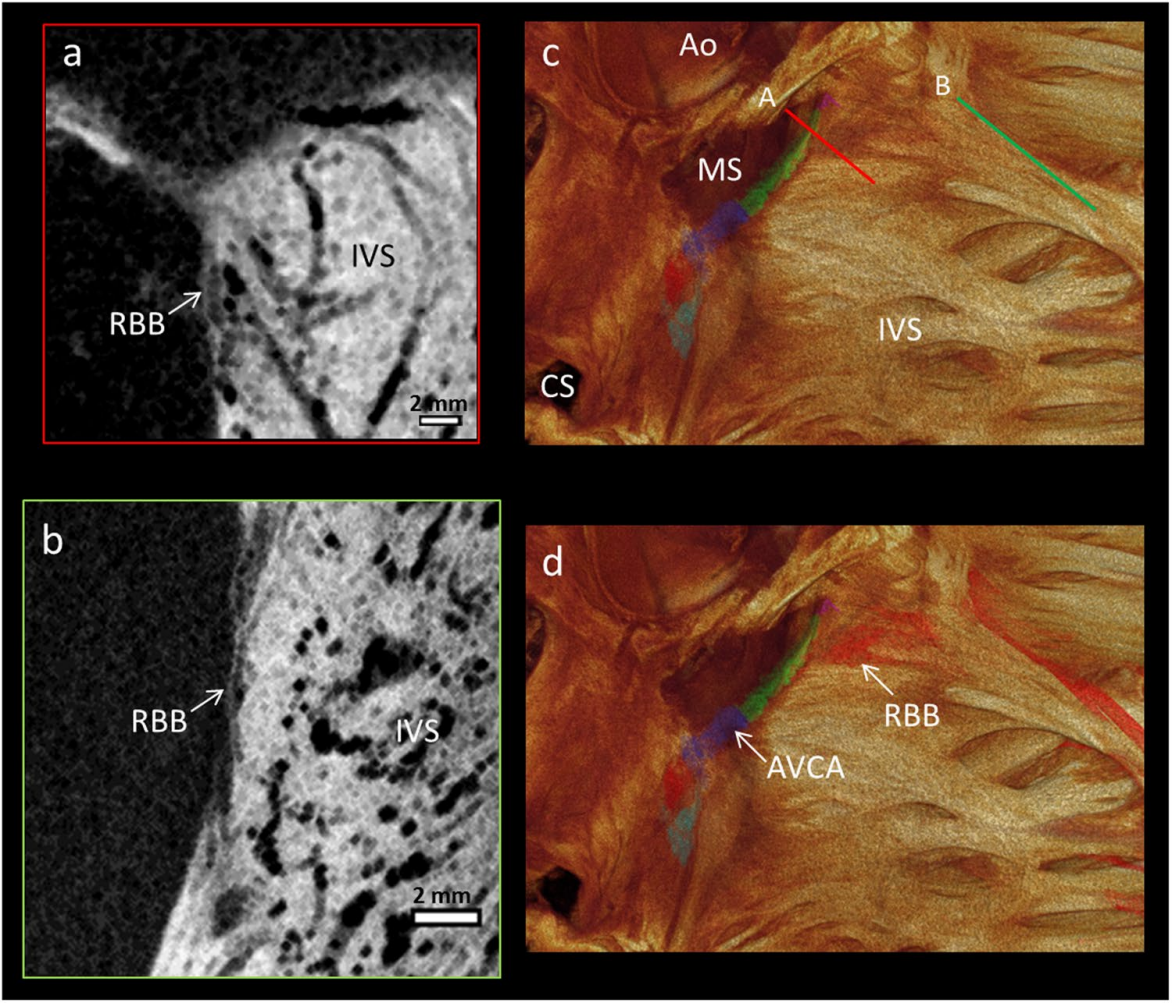
Segmentation of the right bundle branch. The high resolution (73 × 73 × 73 µm^3^) micro-CT data from the whole human heart, showing the 3D extent and position of the segmented right bundle branch (red in panel d) on the lateral aspect of the interventricular septum (**c**,**d**), as viewed from within the right ventricular cavity. Illustrative long-axis micro-CT sections showing the proximal (**a**) and distal (**b**) aspects of the right bundle branch. From the view in (**d**) a part of the so-called dead-end tract at the anterior/rostral extent of the atrioventricular conduction axis can be identified (purple). The position of the cross-sections (**a**,**b**) are shown in the 3D volume rendering in (**c**). Ao- Aortic root, AVCA- atrioventricular conduction axis, CS- coronary sinus, IVS- interventricular septum, MS- membranous septum, RBB- right bundle branch.

**Figure 5:**
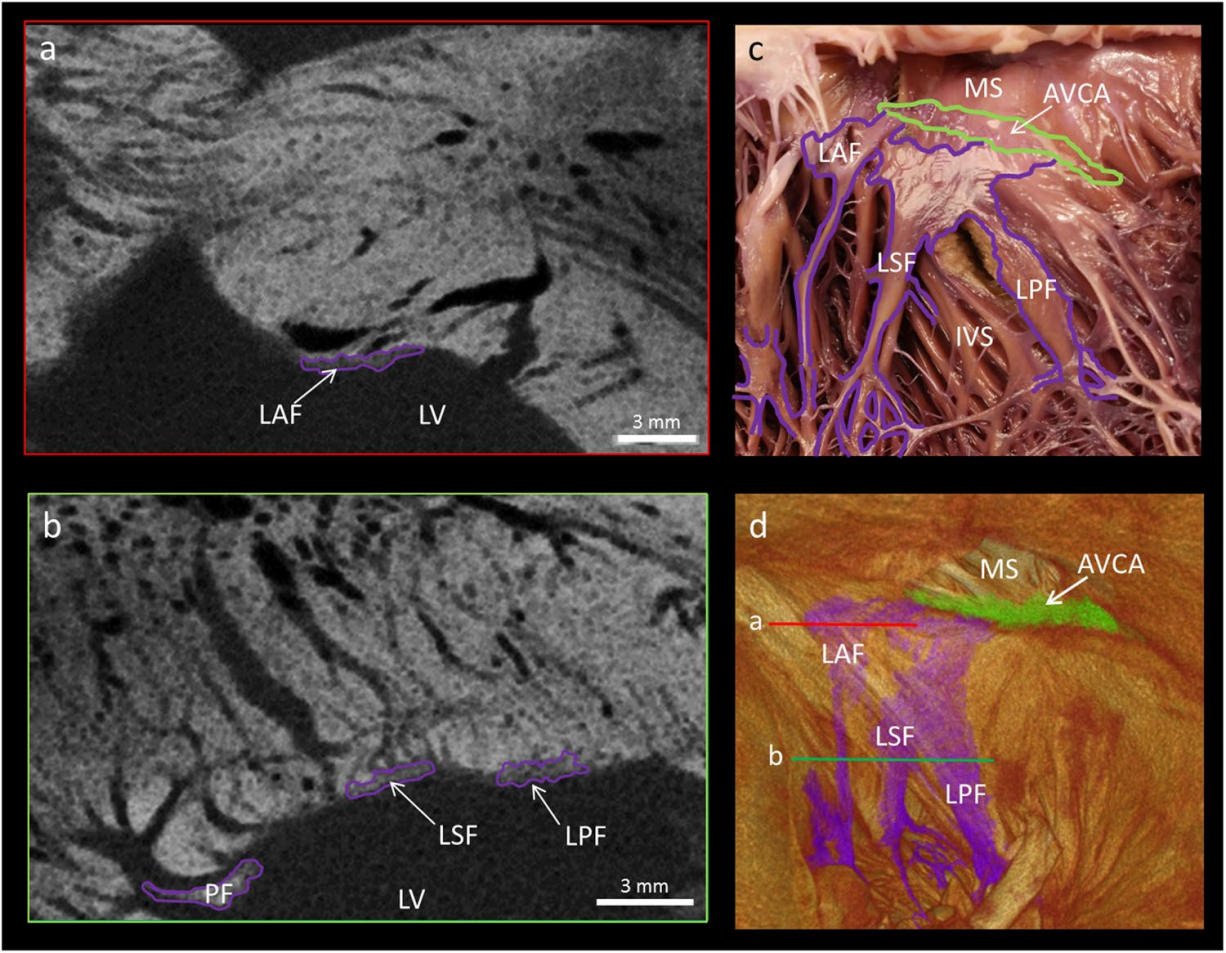
The fascicular arrangement of the left bundle branch resolved by micro-CT. This figure demonstrates high resolution (73 × 73 × 73 µm^3^) micro-CT data from a whole human heart. Panels a and b show short axis micro-CT sections of the ribbon-like anterior, septal and posterior fascicles in the planes indicated in the 3D segmentation (**d**). A comparison between the visual anatomy in a macro photograph and the segmented atrioventricular conduction axis and left sided bundle branches of the same heart is shown in panels c and d. AVCA- atrioventricular conduction axis, IVS- interventricular septum, LV- left ventricular cavity, LAF- left anterior fascicle, LSF- left septal fascicle, LPF, left posterior fascicle, MS- membranous septum, PF- Purkinje fibre.

Analysis showed the atrioventricular conduction axis, comprising the compact node, penetrating bundle, branching bundle, and the ventricular bundle branches, to have pixel values between those of the high-attenuating working myocardium and those of the low-attenuating connective tissue (Fig. 3 and supplementary Fig. S3). It also proved possible to differentiate the inferior nodal extension of the compact node, and to show the continuation of the axis distally as the dead-end tract (Fig. 3 and supplementary Fig. S3). We validated the micro-CT images by performing Masson’s trichrome staining on a block of conduction axis tissue (Fig. 3a,b). In Fig. 3e, we show a short axis volume rendering viewed in the attitudinally correct position, after virtual removal of the atrial myocardium. In Figs 4d and 5d we show 3D representations as viewed from the right and left lateral aspects. For the majority of their course, the penetrating and branching aspects of the axis could be objectively segmented using automatic techniques. At the inferior nodal extension, where the specialised tissue becomes a fine streak of cells surrounded by transitional tissue, semi-automatic segmentation was used. In these regions, existing histo-anatomical descriptions of the inferior nodal extension, and landmarks such as the nodal artery were necessary to guide segmentation. We have not shown the membranous septum in any of the volume renderings. This structure has very low attenuating properties, and is therefore removed from view by the windowing process. In supplementary Fig. S3, we show serial micro-CT sections without outlines.

### The bundle branches and Purkinje network

The conduction axis, via the right and left bundle branches, continues as the Purkinje fibres of the right and left ventricles. We identified the bundle branches as fine low attenuating ribbon-like structures running down the endocardial surface of the ventricular septum (Figs 4 and 5). The right bundle branch was predominantly traced in the long-axis micro-CT sections (Fig. 4a,b), while the left bundle branch was predominantly traced in short-axis micro-CT sections (Fig. 5a,b).

**Figure 6:**
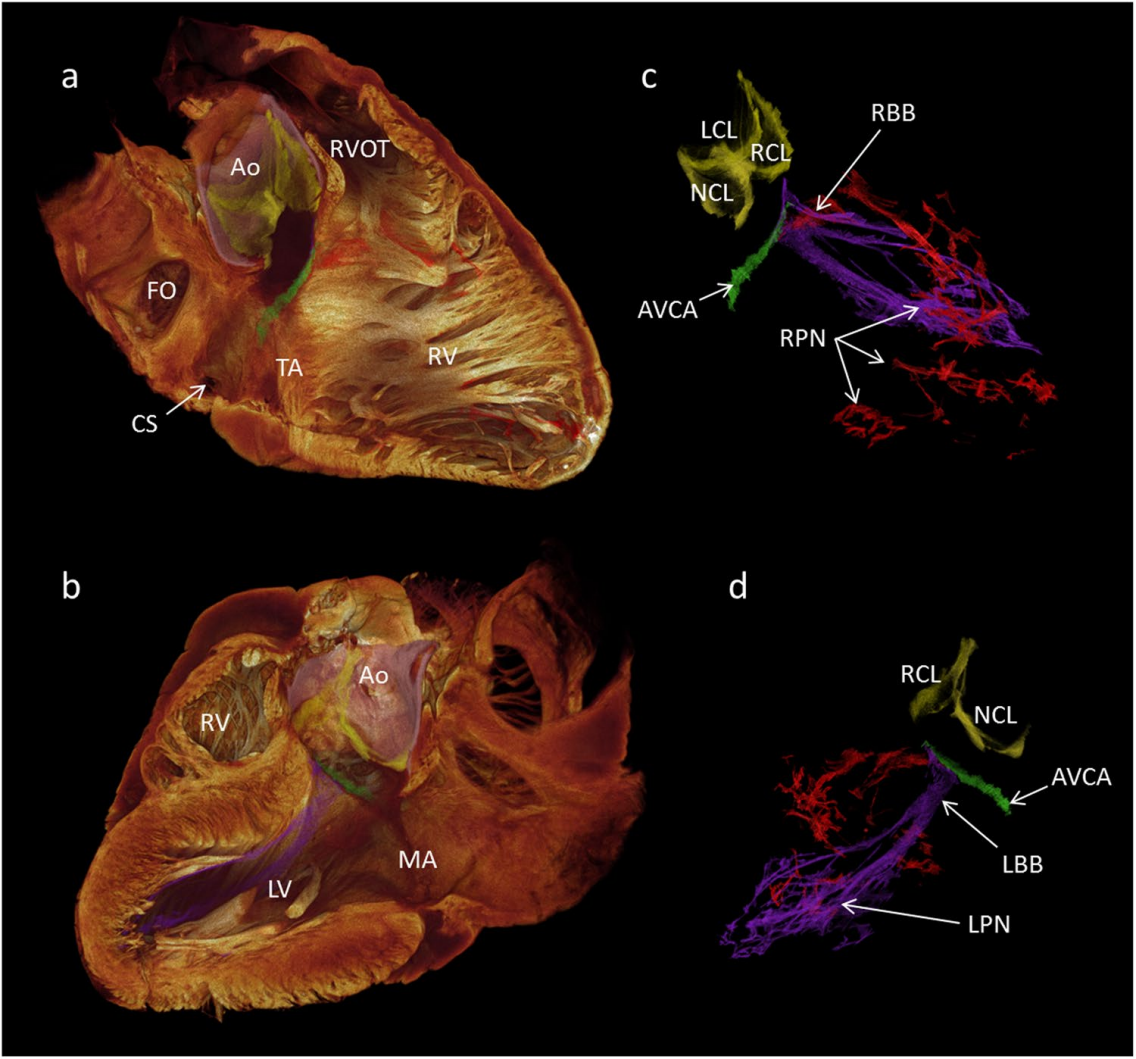
The 3D anatomy of cardiac conduction system in the intact human heart. This figure demonstrates high resolution (73 × 73 × 73 µm^3^) micro-CT data. From a single data set, the cardiac conduction system has been segmented (**c**,**d**) and overlaid on a semi-transparent or ‘ghosted’ rendering of the myocardium and great vessels (**a**,**b**). Panel (a) presents the view from the right side, and panel (b) from the left. The myocardium has been virtually sliced in the longitudinal axis to expose internal structures. Data is presented in the attitudinally correct position for the human, that is, in the upright posture. This 3D dataset is available in the supplementary material as a video (Supplementary Video S1) to demonstrate more clearly the insight that the dataset allows into the anatomical relations between the aortic root, the sinus node, the atrioventricular conduction axis, the atrial and ventricular septa and the bundle branches. Ao- aortic root, AVCA- atrioventricular conduction axis, CS- coronary sinus, FO- fossa ovale, LBB- left bundle branch, LCL- hinge of left coronary leaflet, LPN- left purkinje network, LV- left ventricle, MA- mitral annulus, NCL- hinge of non-coronary leaflet, RBB- right bundle branch, RCL- hinge of right coronary leaflet, RV- right ventricle, RVOT- right ventricular outflow tract, RPN – right Purkinje network, TA- tricuspid annulus.

**Figure 7:**
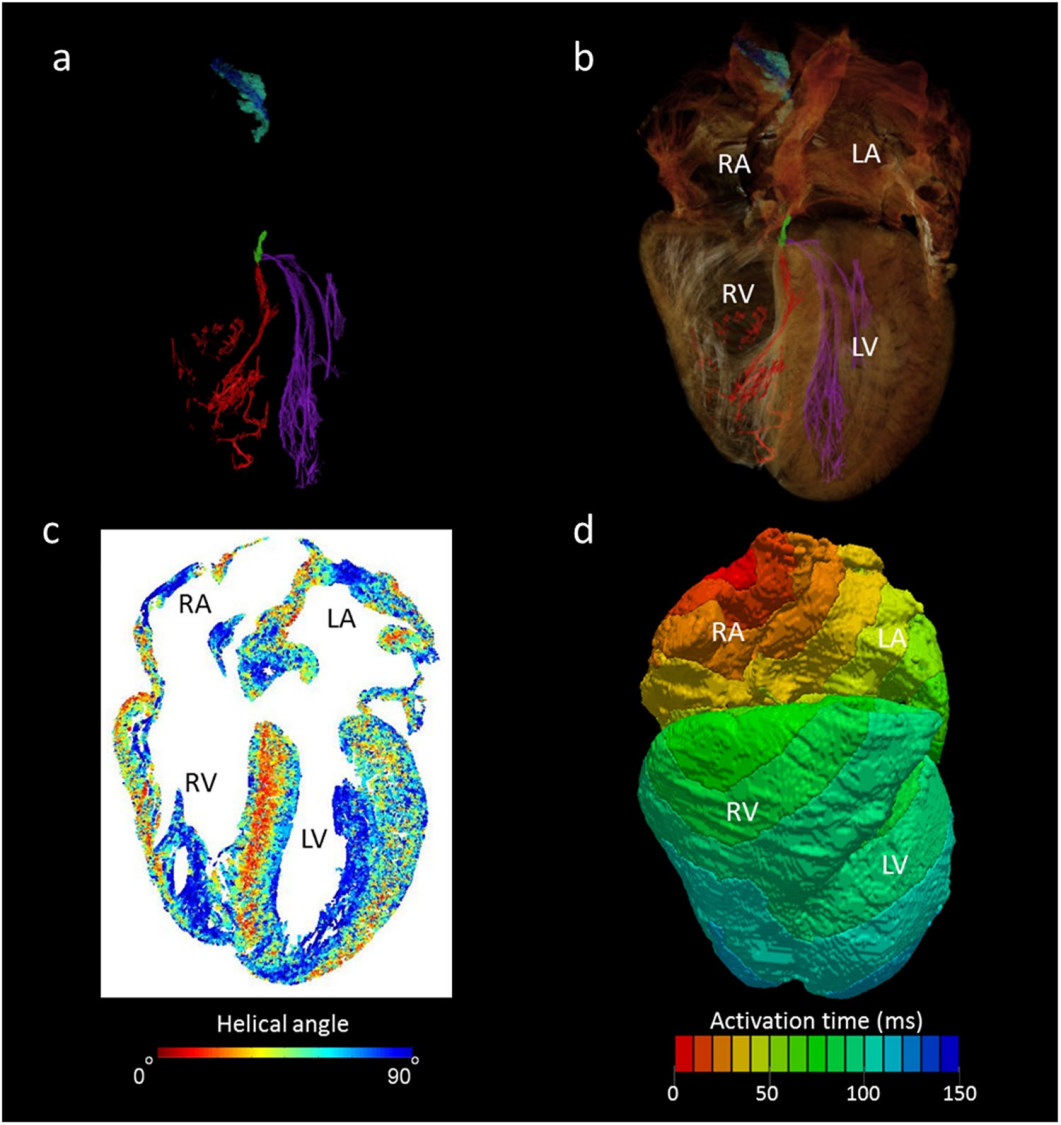
The utility of whole heart micro-CT data in mathematical modelling of cardiac depolarisation. Panel (a) shows the conducting tissue segmented from human whole heart micro-CT dataset; sinus node (blue), paranodal area (turquoise), atrioventricular conduction axis (green), the right (red) and left (purple) Purkinje networks. Panel (b) places the segmented conduction system in the anatomical context of the surrounding myocardium. Panel (c) shows a four-chamber view of cardiomyocyte orientation in which the absolute helical angles derived from the CT dataset are coded in colour (see colour map). Panel (d) is an illustrative isochrone map of cardiac depolarisation seeded from the sinus node. The model incorporates the anatomically accurate geometry of the myocardium and the accurate disposition of the conduction system with the electrical properties of these various regions derived from published electrophysiological measurements. IAS- interatrial septum, IVS- interventricular septum, LA- left atrium, LV- left ventricle, RA- right atrium, RV- right ventricle.

Proximally, the right bundle branch was seen to project anteriorly onto the interventricular septum as a superficial narrow ribbon (Fig. 4a,d), which then descended the septum running on the superficial and deep surfaces of a major free-running trabeculation (Fig. 4b,d). Following the longitudinal axis of the trabeculation, distally the bundle became continuous with the Purkinje network, giving branches to the papillary muscles and supplying the apical region (Figs 4, 6 and 7). The right bundle branch could not be traced continuously for its entire length. This is probably due to insufficient spatial resolution.

The left bundle branch was identified proximally as a broad sheet-like structure draped over and down the left basal surface of the muscular ventricular septum. When traced distally, the left bundle branch took on a tri-fasicular appearance, permitting identification of the anterior, septal and posterior fascicles (Fig. 5). These wide ribbon-like structures running on the endocardial surface, then gave rise distally to the Purkinje fibres, which extend to supply the papillary muscles and the apical region (Figs 5, 6 and 7a,b). A macro photograph of the region in the same heart is shown in Fig. 5c. The specialised conducting tissue cannot be recognised as distinct from the surrounding trabecular structures in such a photograph, but when using the segmented micro-CT data as a guide, the positions of the conducting fascicles and corresponding Purkinje fibres can be indicated. Voxels belonging to the bundle branches and Purkinje networks were selected using semi-automatic segmentation. The Purkinje fibres in this region of the human heart run on the endocardial surface and have many free-running elements (Figs 6 and 7a,b).

### The position of the atrioventricular conduction axis in the intact human heart

Figure 6 shows the 3-dimensional spatial relationship of the conduction axis with the aortic valve. The same data is presented as a rotating 3D video in Supplementary Video S1. In Fig. 6a and b the segmented cardiac conduction system has been integrated with the working myocardium. The renderings have then been sliced longitudinally to expose the internal features of the right and left chambers. The complete segmented datasets without the myocardium are presented in Fig. 6c and d. In this heart, the non-branching bundle extends to within 6.2 mm of the non-coronary leaflet of the aortic valve, while the branching bundle is 6.5 mm away from the nadir of the right coronary leaflet. The dead-end tract approaches to within 15.1 mm of the hinge of the left coronary aortic valvar leaflet. The Purkinje networks run on the endocardial surface with free-running elements within the ventricular lumen. The size and shape of the networks are influenced by the shape and dimensions of the corresponding ventricular cavities. The right ventricular network appears relatively sparse, and the elements span large distances. The left ventricular network is denser, has shorter free-running elements, and takes on a cone-like appearance. We recognise, nonetheless, that this is not a complete representation of the Purkinje networks.

### Computer modelling of electrical depolarisation in the human heart

To confirm the suitability of high resolution micro-CT data for the generation of mathematical models of electrical depolarisation; all major elements of the human cardiac conduction system, along with the surrounding working myocardium from the same heart, were converted into a finite element mesh (Fig. 7a,b). This mesh was then incorporated into an electro-physiologically accurate mathematical computer model of electrical activation (Fig. 7d). The model showed physiological activation of the atria, with the electrical stimulus originating from the sinus node and propagating towards the left atrium and atrioventricular node respectively (supplementary Fig. S4). Preliminary studies of the ventricular activation pattern have highlighted the influence of the extensive and complex Purkinje networks showcased in the anatomical dataset. Further work is now required to incorporate the additional morphological detail of the Purkinje fibre network available in this dataset into an accurate cellular model of the Purkinje system.

**Figure 8:**
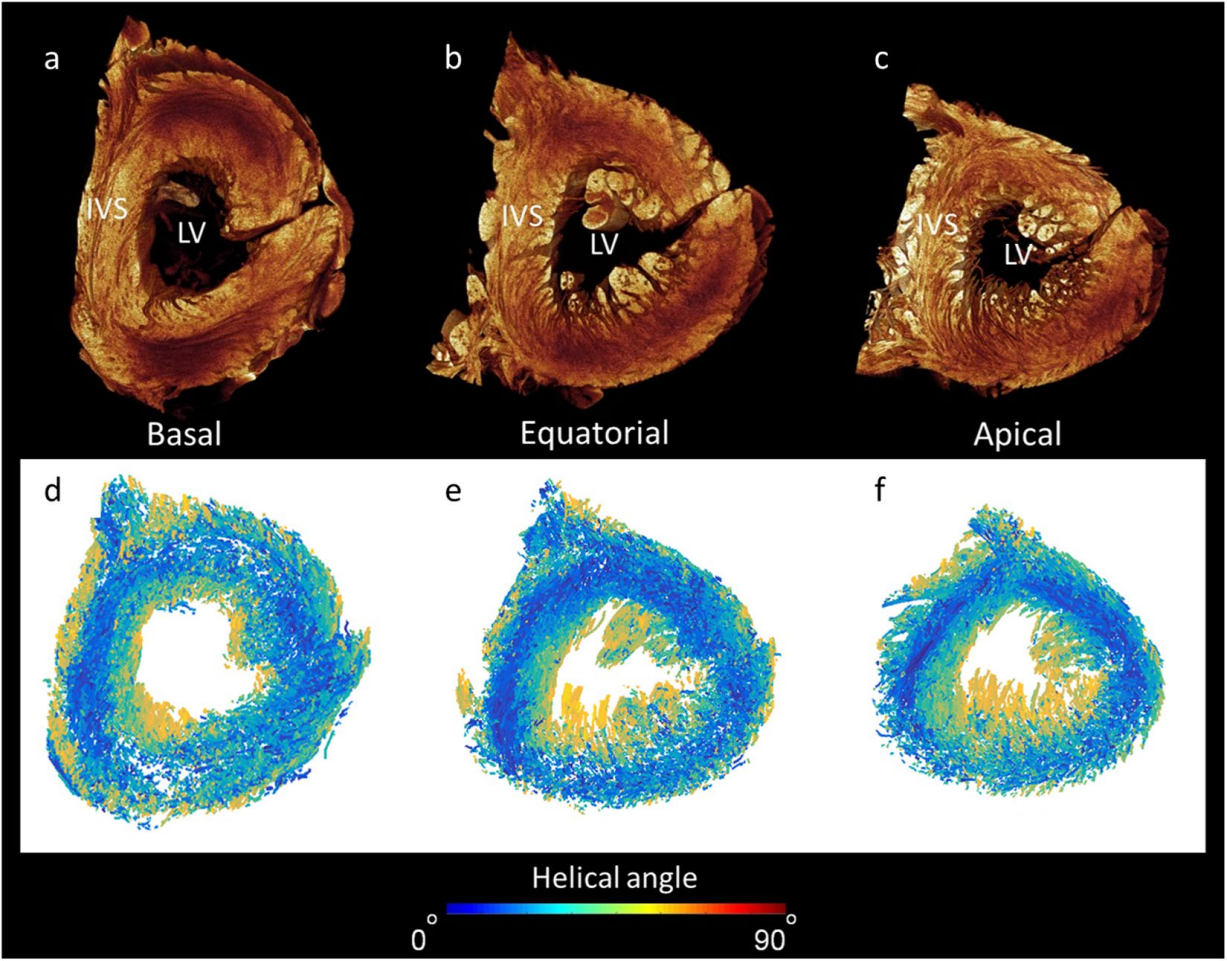
Extraction of cardiomyocyte orientation in the human left ventricle. Panels a–c show volume renderings in short-axis view taking from the basal, equatorial, and apical regions. Panels d–f show corresponding cardiomyocyte orientation in which the absolute helical angles derived from the CT dataset are coded in colour (see colour map). Note the right ventricular free wall is removed from view. IVS- interventricular septum, LV- left ventricular cavity.

The micro-CT dataset, nonetheless, contains additional important information that will improve the prediction of the wave of cardiac depolarisation by computer models. Using Eigen-analysis of the 3D structure tensor (see methods), cardiomyocyte orientation was extracted in the whole human heart (Heart 1) (Figs 7C and 8). The results presented in Fig. 7c show a helical angle colour map in the 4 chamber view: the map is formed from a z-stack of 15 individual tomograms. In the atrial walls, myocytes are aligned along the longitudinal axis of major muscle bundles. A more heterogeneous pattern of myocyte orientation appears within the atrial septum. In the ventricular walls, the myocytes appear as a complex meshwork of myocyte chains (Fig. 8). A clear transmural helical pattern, with a central circumferential zone, was observed across the septum, while a weaker helical pattern, although still prominent, was seen across the left ventricular free wall (Figs 7c and 8). The papillary muscles within the right and left and ventricular cavities, in contrast, contain myocytes running parallel with their long axes. To our knowledge this is the first time the orientation of cardiomyocytes has been extracted from the whole intact human heart at spatial resolution approaching that of the single cell. Virtual models of human cardiac depolarisation will need to take this new quality of information into account in future development.

## Discussion

In this study, we show that contrast enhanced micro-CT provides a non-destructive method with which to produce high-resolution maps of the 3D disposition of the cardiac conduction system in the human heart. Such a technique for imaging the 3D morphology of the human heart will have several important benefits. It has the potential to improve our understanding of the morphological variation between healthy, ageing, diseased, and congenitally malformed hearts. It will facilitate the creation of anatomically and biophysically-detailed mathematical models of the heart. These models will permit creation of 4D simulations of conduction in normal and abnormal electrocardiograms, and allow exploration of micro-structural substrates for arrhythmias, and identification of ablation targets. Such models can also be implemented in drug discovery. The models can be manipulated at the algorithm level to mimic the effects of drugs: for example, the effects of specific pharmaceuticals on ion channel activity.

Over and above this, the knowledge provided can help in refining techniques for implantation of prosthetic valves. It is relatively common for patients undergoing aortic valve replacement to develop arrhythmogenic complications, requiring additional treatment such as the implantation of a pacemaker^39^. The precise 3D positioning of the valvar prosthesis is, therefore, a crucial factor. When the implant is positioned below the native valve, an increase in the frequency of cardiac conduction defects is observed^40^. This observation is not unexpected when the 3D model and Supplementary Video S1 is examined. If subsequently proven to be of comparable value in congenitally malformed hearts, the technique could guide the planning of reconstructive surgery. This would be an important advance, particularly in those complex lesions such as hearts with isomeric atrial appendages, where uncertainty still exists regarding the location of the conduction tissues.

We recognise that, in many respects, the data we present is confirmation of previous studies^22, 41, 42^. It is the first, however, to show 3D representations of the relationships between the conducting tissues and the surrounding cardiac anatomy, and the first to show the three major components of the conduction system in a single intact human heart. This data therefore offers a step change in 3D visualisation of human cardiac anatomy.

In this regard, the findings are comparable with previous studies in the human heart^22, 43, 44^. As is known from investigation of animal hearts^12, 23^, the sinus node extends along a considerable portion of the intercaval region. Significant variation in sinus node length has been observed in the human heart. Sanchez-Quintana *et al*. recorded lengths ranging from 8 mm to 21.5 mm, and showed that the sinus node was not proportional to heart size, nor to the length of the terminal crest. This is consistent with the present study, but contrary to many traditional descriptions and textbook depictions, in which the node is described as a concentrated nodule of cells. In this study, we were also able to trace, segment, and show the 3D extent of a region believed to correspond to the paranodal area. This region may act similarly to the transitional tissue observed in the region of the compact node, facilitating the spread of depolarisation between different tissue types^22, 44^.

We have revealed multiple myocyte projections that emerge from the sinus node to enter the atrial working myocardium (Fig. 2 and Supplementary Figs S1 and S2). These projections had similar voxel values to the paranodal areas, and could be potential exit pathways from the sinus node. The presence of such pathways has been debated for many years^33, 34, 44^. Optical mapping in the region suggests that there are distinct sites of breakthrough for activation of the atrial myocardium^34, 35, 45^. In a histological study by Sanchez-Quintana *et al*. (2005) extensions were found to project from the head, body and tail regions of the sinus node. Consistent with those findings, we resolved projections running towards the endocardial region of the terminal crest, and also towards the epicardial surface. Our high-resolution data suggest that direct connections towards the pectinate muscles may also exist (Fig. 2 and Supplementary Fig. S1). The paranodal area has the potential to act as a pacemaker, and many tachycardias in man have been observed to originate from the region of the terminal crest^14, 22^. In this regard, our data has potential use in computer modelling studies investigating the function of the paranodal area, and the role of these apparent exit pathways, previously shown only in 2D histological sections^44, 45^.

Consistent with Tawara’s original histological description of the atrioventricular conduction axis^26^, our 3D representation confirms that the conduction axis originates at the apex of the triangle of Koch. When traced distally, the axis follows around the circumference of the virtual basal ring of the aortic valve, also known as the ‘echographic annulus’^46^, to the region of the so-called dead-end tract^47^. The presented longitudinal micro-CT tomograms, therefore, correspond well with the seminal histological studies of Tawara^26^, Davies^48^ and Truex^49^. The 3D rendering in our study, however, is superior in that it shows the connections to the bundle branches. Although we can identify all its components, our technique does not differentiate the various sub-compartments in terms of function. Functional distinction requires appropriate staining and immunolabelling of marker proteins^42^.

Our current findings are also in keeping with previous studies of the bovine heart made using immunohistochemistry^50^ and India ink injection^4^. Virtual re-slicing in multiple directions to aid the accurate segmentation of the bundle branches and Purkinje network, as used in the present study, is easily achieved in our 3D isometric micro-CT data. Such re-slicing is not possible by standard sectional histology. The shortcomings of traditional techniques explain the disparity between previous anatomical descriptions of the region as described by Demoulin *et al*.^51^.

The 2D and destructive nature of traditional techniques (histology and dissection) leads to an underestimation of the free-running elements of the Purkinje network. Although the free-running elements are resolved using micro-CT^12^, the depiction presented in the current study is not complete. Based on previous depictions in rabbit^10^, made at even higher resolution, we suggest that although all ‘connecting branches’ are resolved, this is not the case for the ‘terminal branches’. Distally, Purkinje fibres lose their fibrous sheaths^4^, and this finding could explain the reduced differential attenuation observed in some areas of the network. Contrary to many text-book depictions, but consistent with previous immunohistochemical studies^50^, we found no evidence of intramural Purkinje fibres in the human heart.

Although experimental data exist on the remodelling of the conduction system in disease at a cellular and protein level^52^, little is known on how the 3D morphology is affected. This is well illustrated by current mathematical models of electrical depolarisation. Multi-cell models incorporate highly detailed functional information, but model fidelity is confounded by simplified morphological data with gross resolutions^16, 18–20^. Such models often integrate morphological data derived from histology^18^, and in some cases from disparate species^19, 20^. This highlights the importance of the whole heart high-resolution data presented in the current study. Our initial modelling studies show that the large file size, and fine structural complexities, however, makes the incorporation of high-resolution micro-CT data, both computationally and theoretically challenging. This highlights a new research challenge for the modelling community. While providing new challenges, such high resolution 3D data provides a stepwise improvement in the structural geometries available to groups working on mathematical models of cardiac depolarisation.

It has long been accepted that conduction velocity is faster along the longitudinal axis of a cardiomyocyte chain than across its short axis^21^. The path and velocity of the electrical impulse is therefore strongly influenced by cellular orientation. The ability to extract 3D cardiomyocyte orientation at spatial resolutions approaching the single cell, therefore, promises to improve further the fidelity of cardiac modelling. Cellular orientation in specific regions has been described previously^22, 23, 53, 54^. Given its finer resolution, however, micro-CT data is inevitably more complex than the equivalent data from diffusion tensor magnetic resonance imaging. Our data shows the ventricular myocardium to constitute a complex meshwork of cardiomyocyte chains (Fig. 8). The presented method provides a technique to resolve the controversies regarding the arrangement of the cardiomyocytes within the ventricular cone^2, 36^.

Micro-CT has already shown its potential use in virtual archiving of explanted cardiac tissue, in particular congenital malformations^55^. The present study offers a technique to provide the first high resolution 3D anatomical descriptions of the conduction system in such malformations. When the data is presented as 3D images or 3D printed models, it will inform discussions between medical teams and their patients, and aid the education of medical and surgical trainees. The benefit of access to anatomically accurate printed models is already the subject of a clinical trial in the planning of paediatric cardiac surgery (http://www.chop.edu/news/childrens-hospital-takes-part-3dheart-clinical-study)^56^. This technique promises to have considerable impact on the understanding, and strategies, associated with ablation and reconstructive surgery in diseased and congenitally malformed hearts. It should reduce the incidence of conduction abnormalities post-intervention, and, thus, reduce patient morbidity and the need for expensive pacemaker implantation.

## Methods

### Human sample details and ethical approval

Human hearts were obtained post mortem from 4 individuals. The first two hearts were obtained and prepared at the University of Minnesota, USA. They were recovered from organ donors whose hearts were deemed unsuitable for transplantation. Each heart was stopped using cardioplegia, recovered, and transported to the University of Minnesota within 12 hours via LifeSource (Minneapolis, MN). All appropriate consents were obtained for donation for these hearts for research (HH074 and HH059 http://www.vhlab.umn.edu/atlas/histories/ for additional information relating to these specimens). The blocks containing the sinus node and the atrioventricular conduction axis were prepared by Dr. Filip Perde and supplied as frozen samples. Ethical approval was obtained from the National Institute of Legal Medicine, Bucharest, Romania. All subsequent handling and analysis was carried out in accordance with the UK Human Tissue Act (2004).

### Whole heart tissue preparation

The first two hearts, considered to be anatomically normal, were initially flushed with saline solution to remove any residing clotted blood. Next, they were perfusion-fixed with 10% formalin for at least 48 hours to maintain an end-diastolic state, as previously described^12, 57^, and transported to the University of Manchester: a Materials Transfer Agreement was executed and shipping methods followed the guidelines of the Environmental Health & Safety Department of the University of Manchester. The first heart was stained with 7.5% I_2_KI for 14 days, prior to micro-CT scanning^12, 57^. The contrast agent was replaced at day 7. To maintain the chambers in an inflated state for scanning, the heart chambers were filled with warm agarose solution, which becomes a stable gel at the room temperature used during scanning. The second heart was scanned without iodine contrast enhancement and then processed for histological serial sectioning.

### Preparation of blocks containing the Sinus node and atrioventricular conduction axis

The frozen tissue blocks containing the samples were thawed and fixed in 10% neutral buffered formalin (pH 7.4; Sigma Aldrich) at room temperature for two weeks. The sinus node sample was then submerged in 7.5% aqueous iodine-potassium iodide (I_2_KI) contrast medium for 1 week, prior to micro-CT scanning (see below). In preparation for histology, iodine was leached from the tissue and parts of both samples were snap frozen in isopentane cooled in liquid nitrogen. The samples were then stored at −80 °C. Samples were sectioned at a thickness of 25 to 50 µm, sections of interest were stained with Masson’s trichrome. Full descriptions can be found in the data supplement.

### Micro-Computed Tomography

Micro-CT scanning was carried out using the Nikon Metris XTEK 320 kV Custom Bay and Nikon XTEK XTH 225 kV systems at the Manchester X-Ray Imaging Facility, University of Manchester, as previously described by Stephenson *et al*., (2012). The samples were cleared of fixative and excess contrast medium by washing in distilled water, and then immobilized in a plastic holder to prevent movement during the imaging process. Scans were acquired with X-ray energies ranging from 85–160 kV. 360° scans were performed and data was collected from between 2000 and 3142 projections with two frame averaging. A tungsten target was used for all scans, with a 1 mm and 0.25 mm aluminium filter for the whole heart and sinus node prep respectively. Total scan times were approximately 50 minutes. Data was reconstructed using filtered back-projection, resulting in tomographic image data with an isotropic voxel size of 73 × 73 × 73 µm^3^ for the whole heart data and 28 × 28 × 28 µm^3^ for the sinus node sample.

### 3D anatomical reconstruction based on micro-computed Tomography

Data sets were initially viewed, cropped, and manipulated using ImageJ 1.45i (http://rsbweb.nih.gov/ij/). Regions of interest were identified using known landmarks, as used in dissection and histological studies of the cardiac conduction system. 3D reconstructions of the regions of interest were then created in Amira 5.33 using the volume rendering and segmentation techniques previously described^12, 13, 57^. Full descriptions can be found in the data supplement.

Volume rendering and segmentation techniques can be combined. In this study we present volume renderings of the intact human heart and the right atrium (sinus node sample), in which segmentations of the major regions of the cardiac conduction system are overlaid. Although histologically validated, the 3D digital reconstructions represent our hypothesis of the relevant anatomical features of the cardiac conduction system.

### Extraction of 3D cardiomyocyte orientation from micro-computed tomography data

The original 2D micro-CT image series was imported into MatLab Release 2011b (The MathWorks Inc., Natick, Massachusetts, USA 2011) for processing. The micro-CT images had excellent attenuation contrast allowing discrimination between the cardiomyocyces and extracellular spaces. The structure tensor approach is a mathematical approach developed to estimate the orientations of the long axis of the myocytes by utilizing eigen-analysis, and has been demonstrated as a comparable approach with diffusion tensor imaging^58, 59^.

### Mathematical modelling of electrical activation

The computational model was developed by using the segmentation dataset created from the micro-computed tomography reconstruction. The segmentation labels were arranged in a 3-dimensional grid and formed the basis of this virtual human heart. The ten Tusscher^60^ and Countermanche^61^ mathematical models for the human ventricular and atrial tissue were used to simulate the cell electrophysiology for the respective regions. Electrical excitation was manually initiated at the centre of the body of the sinus node and propagated through the tissue. A full description can be found in the data supplement.

### Data availability statement

The datasets generated during and/or analysed during the current study are available from the corresponding author on reasonable request.

## Electronic supplementary material

Supplementary material

Supplementary Video S1
