# Supplementary material for: High resolution 3-Dimensional imaging of the human cardiac conduction system from microanatomy to mathematical modeling
Source: Sci Rep. 2017 Aug 3;7:7188. doi: 10.1038/s41598-017-07694-8 (PMC5543124; doi:10.1038/s41598-017-07694-8)
Supplement: Supplementary file 1 — Supplementary material [file 41598_2017_7694_MOESM1_ESM.doc]

### High resolution 3-Dimensional imaging of the human cardiac conduction system from microanatomy to mathematical modeling

Robert S. Stephenson^1,2^†, Andrew Atkinson^3^†, Petros Kottas^4^, Filip Perde^5^, Fatemeh Jafarzadeh^3^,

Mike Bateman^6^, Paul A. Iaizzo^6^, Jichao Zhao^7^, Henggui Zhang^4^, Robert H. Anderson^8^, Jonathan C. Jarvis^2^§*, Halina Dobrzynski^3^§*

### ^1^ Comparative Medicine Lab, Department of Clinical Medicine, Aarhus University, Denmark; ^2^ School of Sport and Exercise Sciences, Liverpool John Moores University, UK; ^3^Faculty of Biology, Medicine and Health, University of Manchester, UK; ^4^School of Physics and Astronomy, University of Manchester, UK; ^5^National Institute of Legal Medicine, Bucharest, Romania; ^6^The Visible Heart Laboratory, University of Minnesota, Minneapolis, USA; ^7^Auckland Bioengineering Institute, University of Auckland; ^8^Institute of Genetic Medicine, University of Newcastle.

†Joint first authors, §Joint senior authors, *Corresponding authors

**Supplementary material**

**Supplementary figures**


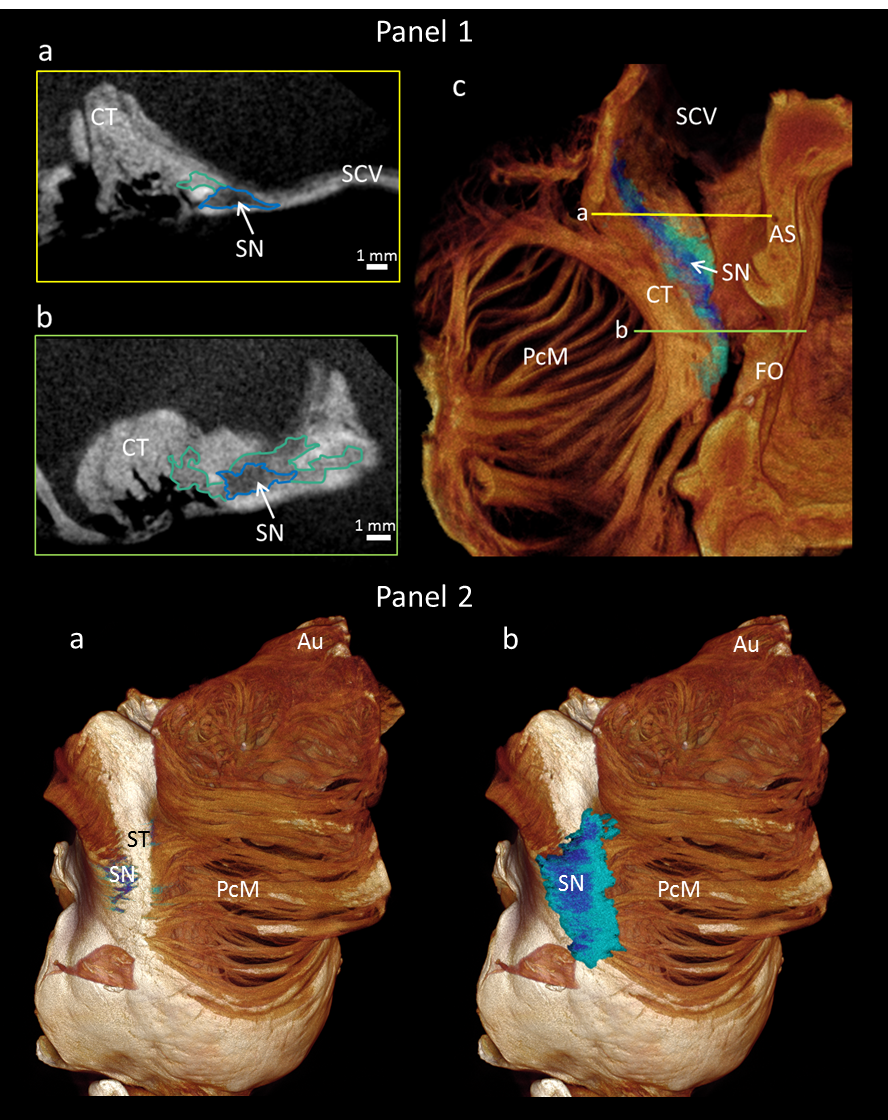


**Supplementary Figure S1. *Objective identification of the human sinus node and paranodal area by micro-CT.*** *Panel 1: high resolution (73 x 73 x 73 µm^3^)* *micro-CT data from an intact human heart. The sinus node (blue) and paranodal area (turquoise) is outlined in short-axis (a, b) micro-CT images. (c) Volume rendering (endocardial view) showing semi-automatic segmentation of the low pixel values corresponding to the sinus node body are shown in blue, pixel values corresponding to the paranodal area are shown in turquoise. Panel 2: high resolution (28 x 28 x 28 µm^3^)* *micro-CT data from a sub-volume preparation of a whole human heart, in this case the area of the right atrium containing the sinus node. Volume rendering (epicardial view) showing objective segmentation (a) of the low pixel values corresponding to the sinus node body are shown in dark blue, pixel values corresponding to the paranodal area are shown in turquoise. (b) Entire segmented volume overlaid onto myocardium. AU- auricle, CT- terminal crest, FO- fossa ovalis, ICV- inferior caval vein, PcM- pectinate muscles, SCV- superior caval vein, SN- sinus node, ST- sulcus terminalis.*


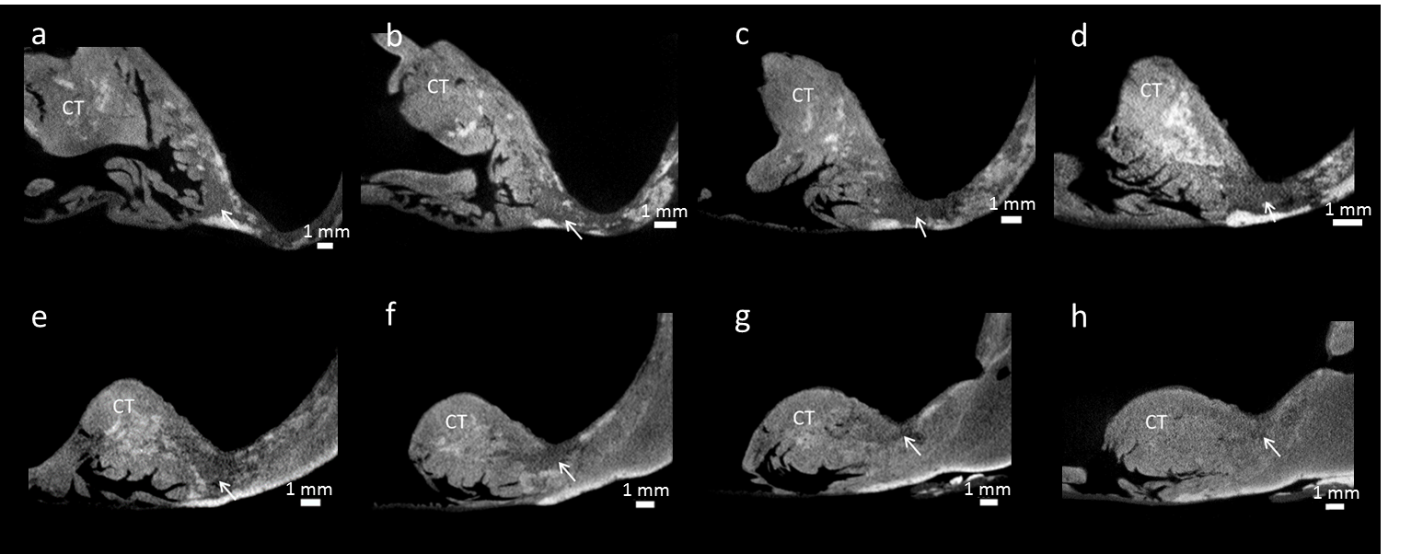


**Supplementary Figure S2. *Virtual serial sections of the human sinus node.*** *This figure demonstrates high resolution (28 x 28 x 28 µm^3^)* *micro-CT data from a sub-volume preparation of a whole human heart, in this case the area of the right atrium containing the sinus node. Serial (superior to inferior) short axis micro-CT images (a-h), illustrating the low attenuating sinus node (arrows), and accompanying low attenuating paranodal projections and islands. CT - terminal crest.*


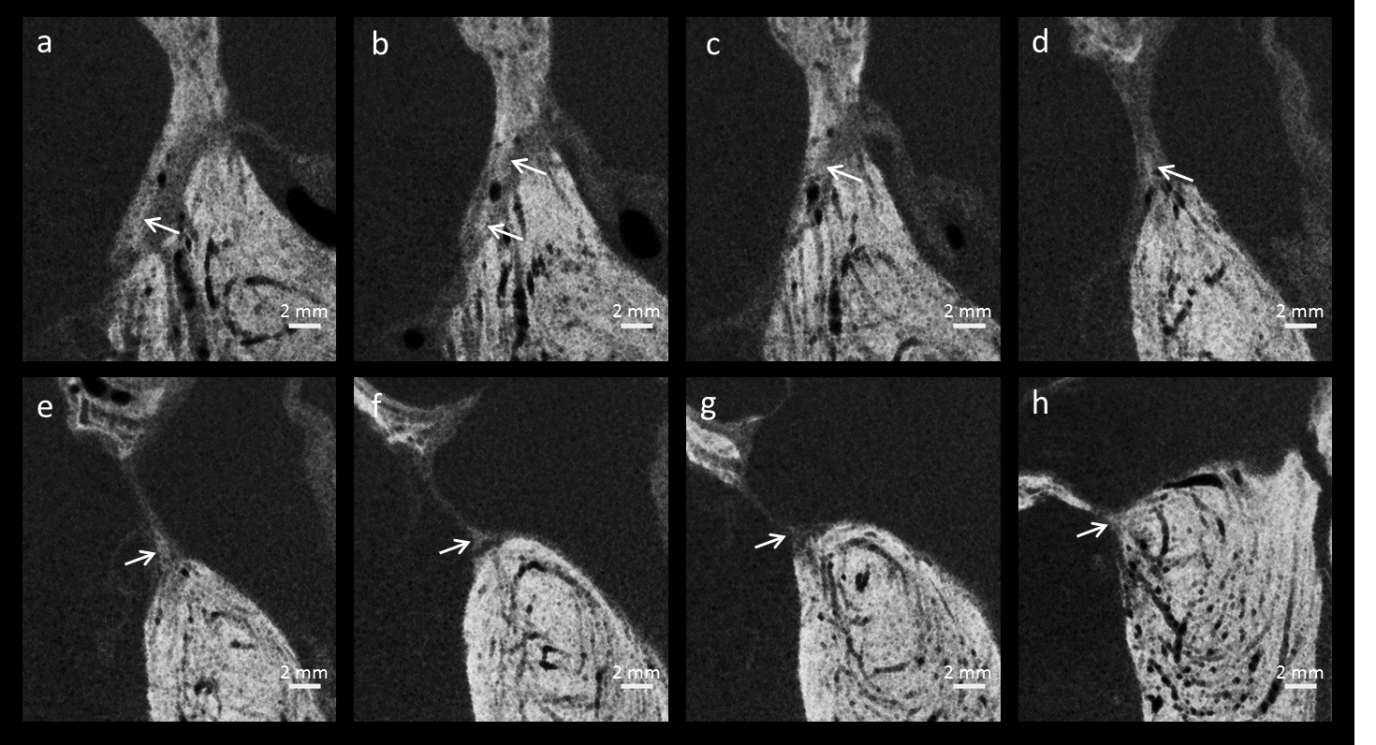


**Supplementary Figure S3. *Virtual serial sections of the human atrioventricular conduction axis.*** *This figure demonstrates high resolution (73 x 73 x 73 µm^3^)* *micro-CT data from an intact human heart. Serial (infero-posterior to supero-anterior) longitudinal micro-CT images (a-h), illustrating the low attenuating atrioventricular conduction axis (arrows). All regions are resolved; Inferior nodal extension (a), compact node (b, c) and inferiorly lying nodal extension (b), penetrating bundle (d,e), branching bundle (f, g), dead-end tract (h).*


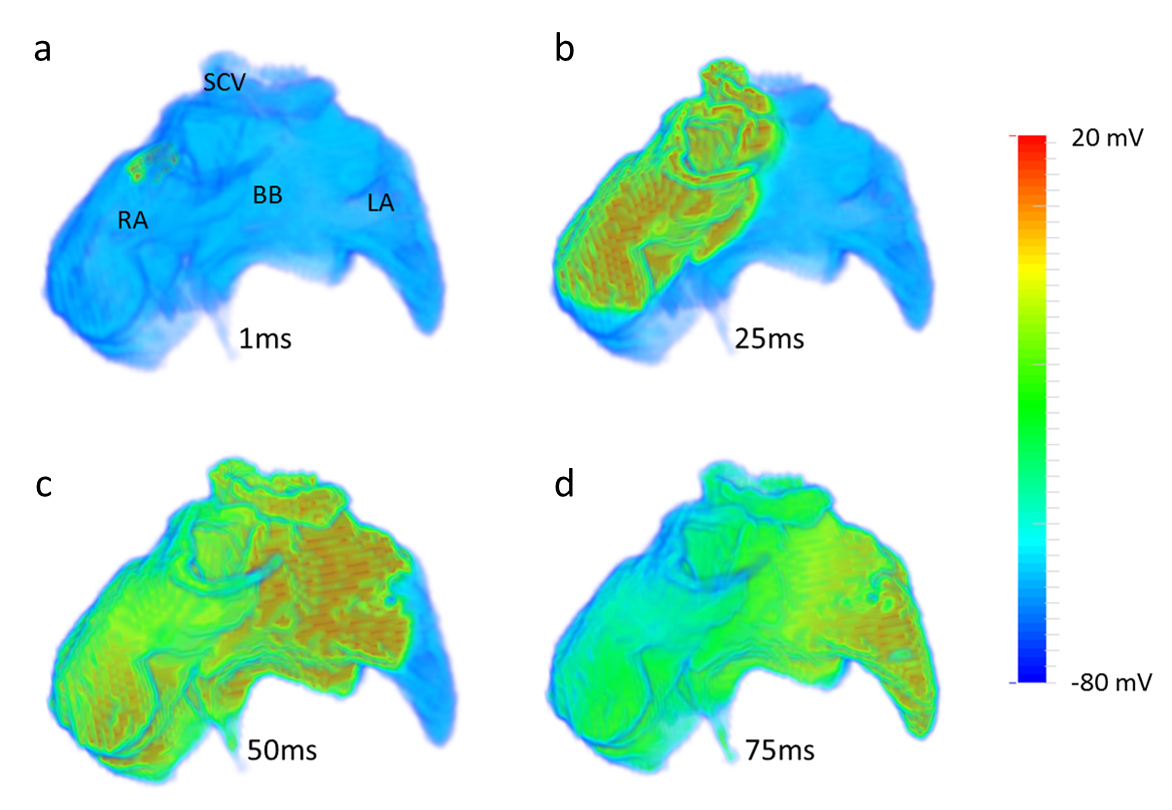


**Supplementary Figure S4. *Mathematical simulation of atrial electrical depolarisation.*** *Panels a-d show the spread of depolarisation at time points of 1, 25, 50, and 75 ms. The sinoatrial node was used as the seed point. Conduction velocity was set to 100 cm/s for the crista terminalis and Bachmann’s bundle region and 60 cm/s for the rest of the atrial tissue.*

**Supplementary methods**

***Whole heart tissue sectioning***

After micro-CT scanning the second unstained intact heart was filled with chilled 2% carboxymethyl cellulose paste. It was then frozen in a mixture of hexane and solid carbon dioxide for 60 minutes. It was sectioned using a Leica CM3600 XP cryomacrotome (Leica Microsystems) at a thickness of 50 µm onto adhesive collection tape and the sections were allowed to dry in air at room temperature. Six serial sections were collected and then 500 µm was discarded (forming each level) until the whole preparation had been sectioned. 950 sections were collected representing the entire volume.

***Histology***

Masson’s trichrome histology stains were prepared in accordance with protocols previously described^1-3^.

Glass slides containing sections and adhesive collection tape containing sections from different regions of the whole heart were fixed in Bouin’s fluid (Sigma Aldrich) overnight and then cleared using three 10 minutes washes in 70% ethanol. They were stained with celestine blue (Sigma Aldrich) for 5 minutes, rinsed in distilled water, stained with Mayer’s hematoxylin (Sigma Aldrich) for 10 minutes, washed in tap water for 15 minutes, stained with acid fuchsin (Sigma Aldrich) for 4 minutes, then rinsed in distilled water until no stain leached from the sections. Sections were then stained with phosphomolybdic acid for 5 minutes (Sigma Aldrich) followed by methyl blue (Sigma Aldrich) for 5 minutes, and then rinsed in distilled water. They were then treated with 1% acetic acid for 2 minutes, followed by dehydration with ethanol, using 70% for 1 minute, 90% for 1 minute and 100% ethanol twice for 2 minutes. They were then washed twice in Histo-Clear (National Diagnostics) for 5 minutes to clear the dehydrant. Coverslips were mounted onto glass slides using DPX mountant (Sigma Aldrich). With this technique, connective tissue was stained royal blue, cardiac myocytes were stained pink/purple and nuclei were stained dark blue/black.

The sections were imaged using Zeiss SteREO Discovery.V8 (Carl Zeiss Microscopy) and Zeiss Imager.Z1 microscopes (Carl Zeiss Microscopy) using Axiovision software (Carl Zeiss Microscopy) and whole heart sections were imaged using a flatbed scanner.

***3D anatomical reconstruction based on micro-computed Tomography***

Data sets were initially viewed, cropped, and manipulated using ImageJ 1.45i (<http://rsbweb.nih.gov/ij/>). Regions of interest were identified using known landmarks, as used in dissection and histological studies of the cardiac conduction system. Once identified 3D reconstructions of the regions of interest were created in Amira 5.33 using the volume rendering and segmentation techniques.

Volume renderings were created in Amira 5.33 using the 3D volume viewer module. In this automatic technique, an opacity curve and corresponding colour map is applied to all voxels within a pre-selected window of voxel values. The resultant 3D volume is rendered using a VRT render mode in which voxels are assigned a level of opacity (0–100%) calculated from their voxel value (voxel values are correspond to the X-ray attenuation coefficients).

Segmentation methods were based on methodologies previously described by the authors^4-6^. In the objective (automatic) method for segmentation the range of voxel values representing the conduction system is recorded, and used to define a range or ‘window’ of voxel values. The ‘magic wand’ tool is then used at the defined window settings to segment the structure slice by slice, that is, to identify contiguous blocks of pixels that correspond to the defined window of attenuation levels. The ‘magic wand’ tool implements a recursive seed fill method of filling 2D graphic images; the selected seed is allowed to grow by selecting adjacent pixels under the constraint of the masking window. Where there was an overlap of pixel values between the conducting tissues and the surrounding tissues which the seed fill algorithm could not recognise, the ‘limit line’ function was used to create a boundary. The limit line provides a boundary which the seed fill algorithm cannot pass. When the divisions between tissue types are extremely fine, it is inevitable that the absorbance values of adjacent pixels overlap, this is a partial volume effect. Although the specialized tissue can be discerned in serial sections, on some occasions the algorithm requires guidance. For example at the inferior nodal extension, where the specialized tissue becomes a fine streak of cells surrounded by transitional tissue, semi-automatic segmentation was used. In these regions, existing histo-anatomical descriptions of the inferior nodal extension, and landmarks such as the nodal artery were necessary to guide segmentation. Pre-existing knowledge of the micro-anatomy of the cardiac conduction system does indeed aid segmentation. Semi-automatic segmentation was performed as previously described^4^. In brief, the region of interest is selected using the ‘paint brush’ tool, and subsequently this selection is windowed based on the previously defined range of voxel values. In both segmentation methods the ‘interpolation’ function can be applied between two selected regions to improve efficiency. The resultant 3D meshes made up of the voxels attributed to the conduction system components can be manipulated and cropped in any orientation and their respective volumes, surface areas and dimensions quantified.

Volume rendering techniques and segmentation techniques can be combined. In this study we present volume renderings of the intact human heart in which segmentations of the major regions of the cardiac conduction system are overlaid.

***Mathematical modelling of electrical activation***

The computational model was developed by using the segmentation dataset created from the micro-computed tomography reconstruction. The segmentation labels were arranged in a three dimensional grid and formed the basis of this virtual human heart. The ten Tusscher^7^ and Countermanche^8^ mathematical models for the human ventricular, atrial and specialised conducting tissue were used to simulate the cell electrophysiology for the respective regions. Electrical excitation was manually initiated at the centre of the body sinus node and propagated through the tissue as described by the following differential equation:


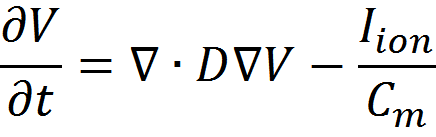


Where V is membrane potential, t is time, D is the diffusion coefficient for the electrical spread throughout the tissue, I_ion_ is the total ionic current and C_m_ is the cell membrane capacitance. This equation was solved using the Finite Difference Method with a space step of 120μm and time step of 2μsec, on a system running Scientific Linux with two Intel Xeon E5 2680v2 processors and 128 GB of RAM. Visual representations of the three dimensional anatomical reconstruction and electrical activation were produced in the Paraview software (Kitware, Inc., Clifton Park, New York, USA), by using the volume renderer, threshold or contour options.

***Details and cause of death of patients studied***

Human hearts were obtained post mortem from 4 individuals. The first heart was from a 60 year old female, in whom the cause of death was bronchogenic metastatic cancer. The second heart was from a 54 year old female, who died due to a cerebrovascular accident. The third heart, which we used to provide a preparation including the sinus node for histologic examination and very high resolution scanning, was from a 36 year old female who died suddenly and unexpectedly. The final heart, which we used to provide material including the atrioventricular conduction axis sample for histologic examination and very high resolution scanning, came from a 19 year old male, in whom the cause of death was a road traffic accident.

**References**

1 Chandler, N. *et al.* Computer three-dimensional anatomical reconstruction of the human sinus node and a novel paranodal area. *Anat Rec (Hoboken).* **294,** 970-979, doi:10.1002/ar.21379 (2011).

2 Chandler, N. J. *et al.* Molecular architecture of the human sinus node: insights into the function of the cardiac pacemaker. *Circulation* **119,** 1562-1575, doi:10.1161/circulationaha.108.804369 (2009).

3 Greener, I. D. *et al.* Molecular architecture of the human specialised atrioventricular conduction axis. *J Mol Cell Cardiol.* **50,** 642-651, doi:http://dx.doi.org/10.1016/j.yjmcc.2010.12.017 (2011).

4 Jarvis, J. C. & Stephenson, R. Studying the microanatomy of the heart in three dimensions: a practical update. *Front Pediatr.* **1,** 26, doi:10.3389/fped.2013.00026 (2013).

5 Nikolaidou, T. *et al.* Congestive heart failure leads to prolongation of the pr interval and atrioventricular junction enlargement and ion channel remodelling in the rabbit. *PLoS ONE.* **10,** e0141452, doi:10.1371/journal.pone.0141452 (2015).

6 Stephenson, R. S. *et al.* Contrast enhanced micro-computed tomography resolves the 3-dimensional morphology of the cardiac conduction system in mammalian hearts. *PLoS ONE.* **7,** e35299 (2012).

7 ten Tusscher, K. H. W. J., Noble, D., Noble, P. J. & Panfilov, A. V. A model for human ventricular tissue. *Am J Physiol Heart Circ Physiol.* **286,** H1573-H1589, doi:10.1152/ajpheart.00794.2003 (2004).

8 Courtemanche, M., Ramirez, R. J. & Nattel, S. Ionic mechanisms underlying human atrial action potential properties: insights from a mathematical model. *Am J Physiol Heart Circ Physiol.* **275**, H301-H321 (1998).
